# Arena-Idb: a platform to build human non-coding RNA interaction networks

**DOI:** 10.1186/s12859-018-2298-8

**Published:** 2018-10-15

**Authors:** Vincenzo Bonnici, Giorgio De Caro, Giorgio Constantino, Sabino Liuni, Domenica D’Elia, Nicola Bombieri, Flavio Licciulli, Rosalba Giugno

**Affiliations:** 1 Department of Computer Science,University of Verona, Strada Le Grazie, Verona, Italy; 2 Institute for Biomedical Technologies, National Research Council (CNR), Bari, Italy

**Keywords:** Non-coding RNA, Database, Network, Data integration

## Abstract

**Background:**

High throughput technologies have provided the scientific community an unprecedented opportunity for large-scale analysis of genomes. Non-coding RNAs (ncRNAs), for a long time believed to be non-functional, are emerging as one of the most important and large family of gene regulators and key elements for genome maintenance. Functional studies have been able to assign to ncRNAs a wide spectrum of functions in primary biological processes, and for this reason they are assuming a growing importance as a potential new family of cancer therapeutic targets. Nevertheless, the number of functionally characterized ncRNAs is still too poor if compared to the number of new discovered ncRNAs. Thus platforms able to merge information from available resources addressing data integration issues are necessary and still insufficient to elucidate ncRNAs biological roles.

**Results:**

In this paper, we describe a platform called Arena-Idb for the retrieval of comprehensive and non-redundant annotated ncRNAs interactions. Arena-Idb provides a framework for network reconstruction of ncRNA heterogeneous interactions (i.e., with other type of molecules) and relationships with human diseases which guide the integration of data, extracted from different sources, via mapping of entities and minimization of ambiguity.

**Conclusions:**

Arena-Idb provides a schema and a visualization system to integrate ncRNA interactions that assists in discovering ncRNA functions through the extraction of heterogeneous interaction networks. The Arena-Idb is available at http://arenaidb.ba.itb.cnr.it

## Background

The availability of omics repositories represents a powerful resource for the discovery of interactions among non coding RNAs (ncRNAs). The association of metadata to ncRNAs allows researchers to exploit their full potential for inferring new molecular functions. Molecular interactions involve several types of entities including Long non-coding RNAs (lncRNAs) and Small non-coding RNAs (sncRNAs), further divided into subclasses shortly called biotypes. According to HUGO Gene Nomenclature Committee (HGNC) [1], the sncRNAs (see Table 1) are classified into various biotypes of short sequences such as Small interfering RNAs (siRNAs), microRNAs (miRNAs), PIWI-interacting RNAs (piRNAs), small nuclear RNAs (snRNAs), small nucleolar RNAs (snoRNAs), and small cytoplasmic RNAs (scRNAs). The lncRNAs have a broader spectrum of functions [2, 3] such as regulation of transcription, RNA processing, nuclear-cytoplasmic transport, translation control and modulation of chromatin structure and are, therefore, a potential new class of cancer therapeutic targets [4]. In addition to these classes of ncRNAs there are other different types of ncRNAs whose role is under discovering. The circular RNA are highly active in brain cells and play an important role in neurodegenerative disease and encoding of proteins [5]. The rigorous characterization of the biological functions of extracellular RNAs (exRNAs) in biofluids is a rapidly growing area of research to monitor diseases with a promising use in diagnostic [6].

**Table 1:** Overview of the major classes of ncRNAs: classification and functional characterization

Symbol	Name	Size	Function
miRNAs	microRNAs	18−24 nt	They act as negative control of gene expression by silencing or catalysing mRNA destabilization.
snoRNAs	Small nucleolar RNAs	70 nt	Conserved nuclear RNA in Cajal bodies or nucleoli where they either function in the modification of snRNA or participate in the processing of rRNA ribosome subunit maturation.
snRNAs	Small nuclear RNAs	100−300 nt	RNA localized in the eukaryotic cell nucleus. They are part of splicesome multisubunit complex which assembles on RNA and carriers out RNA splicing. The snRNAs are classified in different type according of their role.
siRNAs	Small-interfering RNAs	20−25 nt	siRNA derived from much longer double stranded RNA (dsRNA) precursor by DICER ribonucleases and play a substantial role in genetic and epigenetic regulatory.
ceRNAs	Competitive endogenous RNAs	> 200 nt	ceRNAs are transcripts that can crosstalk through their ability to compete for mRNA binding and they act to sequester miRNAs.
circRNAs	Circular RNAs	> 200 nt	circRNAs arise from exons or intronics and may be also translate into protein. Exonic circRNAs are very stable in cell and have specific roles in cellular physiology.
piRNAs	PIWI-interacting RNAs	25−35 nt	piRNAs show specific expression in germ cells. Recent studies suggest that piRNA represents adaptive control mechanisms that protect genomics architectures again transposable elements (TE). Most piRNA are derived from genomic piRNA clusters.
lincRNAs	Long intergenic non-coding RNAs	> 200 nt	Perform various regulatory roles, but the majority remain functionally uncharacterized and typically low abundance and poor evolutionary conservation.
lncRNAs	Long non-coding	> 200 nt	lncRNAs are transcripts that lack RNAs apparent protein coding and are largely heterogeneous and functionally uncharacterized. The increasing evidence began to suggest that they play critical regulatory roles in manu human disease.

In physiological conditions, many biological entities interact with each another and are key regulators of many cellular processes and contribute to a multitude of diseases [7]. Understanding a biological interactions system demands understanding the details of its components, and their interactions. Available public biological resources provide narrowed but systematic overviews of relationship schema among biological entities. For example, an individual miRNA may regulate multiple mRNAs, and in contrast, an individual gene may also be regulated by multiple miRNAs, thus representing a complex network of miRNA-mRNA interactions. More recently, other layers of regulation have added further complexity in regulatory networks. It has been proposed that the binding of microRNAs to their targets can be buffered by transcripts mimicking the sequences of the true targets, therefore protecting them from repression; these transcripts have been called ’competitive endogenous RNAs’ (ceRNAs) [8, 9]. If these ceRNAs possess many miRNAs response elements (MREs) and are expressed at high enough levels, they act to sequester miRNAs [10]. Many existing databases are unified catalogues of annotations, sequences and expression information for human ncRNAs [11–19]. These databases are frequently developed only in the contest of one or few biotypes of ncRNAs and without the integration of diseases associations. Tools such as the ones reported in [20–22] provide an integration procedure which does not verify sequence similarity and is mostly focused on genes, proteins and in some cases miRNAs [14]. Moreover, none of these databases provide an integrated vision of relationships between different ncRNA biotypes and other entities [23, 24]. In this paper, we present a computational framework (Arena-Idb) to realize non-coding RNA-Gene regulatory networks. Arena-Idb addresses the gap of existing methods providing a framework for network reconstruction of ncRNA heterogeneous interactions (i.e., with other type of molecules) and relationships with human diseases which guides the integration of data extracted from different sources via mapping of entities and minimization of ambiguity. Arena-Idb handles knowledge regarding biological products (i.e., information linking transcribed RNA and translated proteins to their corresponding source genes, thus from DNA to RNA or protein, and from RNA to protein) and cross-references (i.e., the mapping between different nomenclature systems). To keep non-redundant sequences it filters the information by comparing cross-link references and sequence similarity using the Cleanup software [25]. Compared to its previous version [26], Arena-Idb provides (i) a mapping procedure for managing entities, (ii) improving the accuracy of the integration process by identifying the sequence entity, (iii) reconstructed data storage and update including seven new sources as Disease Ontology, lnc2cancer, lncACTdb, mir2disease, miRecords, mirSponge, PSMIR, StarBase and TarBase, (iv) a more functional web interface that provides many new features such as, among others, a browser section that allows users to visualize, filter and download data by different criteria; a search section that enables queries also for chromosomal location; and a network visualization system that also allows the download of data in a readable format for Cytoscape import. The Arena-Idb can be accessed or downloaded as whole integration system at http://arenaidb.ba.itb.cnr.it.

## Methods

**Fig. 1:**
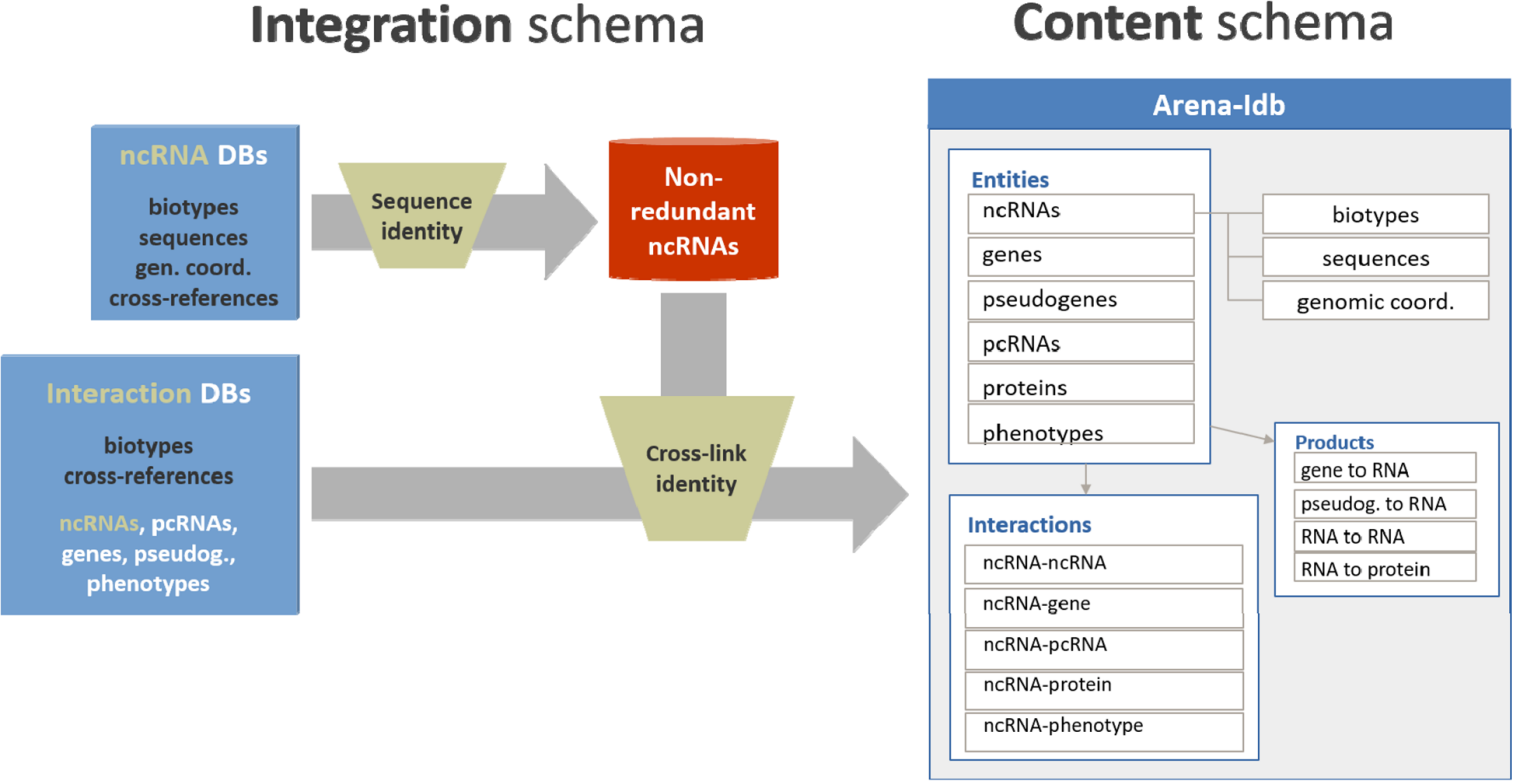
Arena-Idb integration and content overview. On the left, the “Integration schema” which shows the type of data extracted from each type of source used and the processes (sequence identity processing and cross-link identity) performed for to obtain the data stored and integrated into Arena-Idb (Content schema shown on the figure’s right side). The result of the integration process is a comprehensive database collecting information about the objects (genes and their products) and the interactions between ncRNAs and integrated objects

**Fig. 2:**
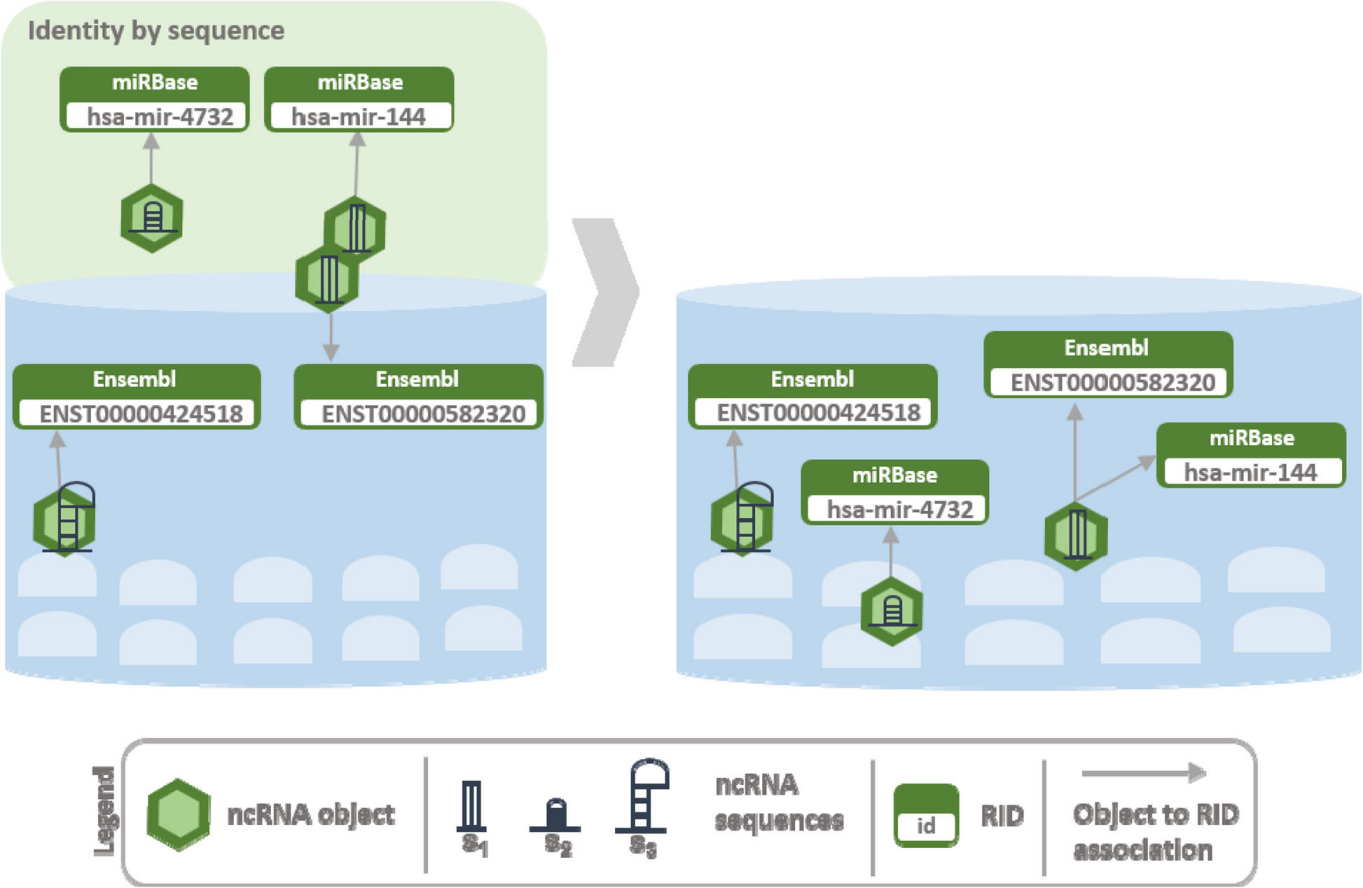
Arena-Idb integration process: identity by sequences. The Figure gives an example of integration performed by sequence identity recognition. Two miRNAs, identified by miRBase symbols, are integrated into a partial state of Arena-Idb that contains two ncRNA, identified by their Ensembl IDs. The sequences of one of the two miRBase miRNAs is recognized in the partial state thus the miRBase symbol is added up to the list of aliases assigned to the miRNA object. Instead, no compatible sequences are found for the other miRBase miRNA. The results of the integration is a collection of three ncRNAs

**Fig. 3:**
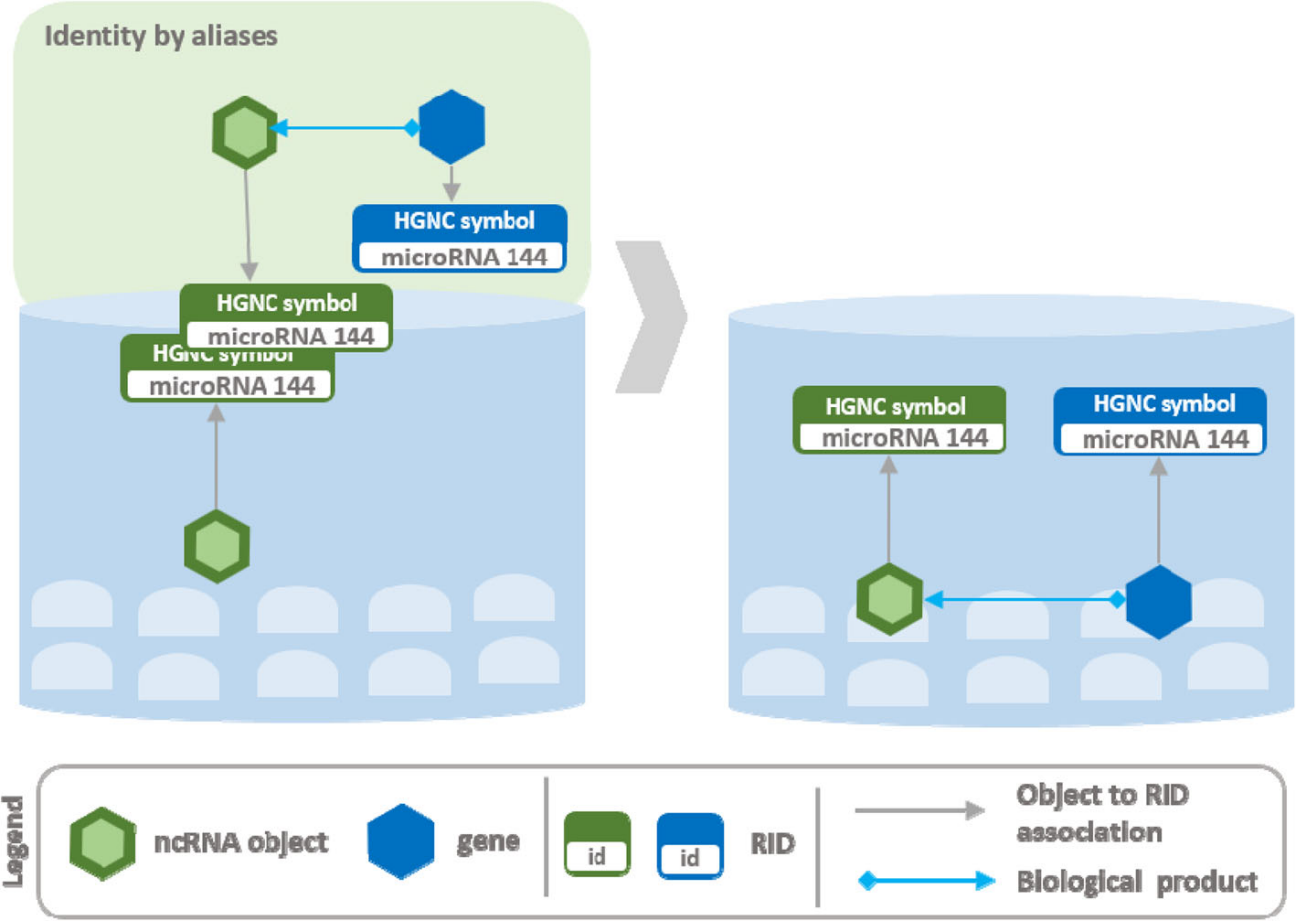
Arena-Idb integration process: identity by aliases, example 1. The Figure gives an example of integration regarding the addition of a ncRNA and a gene into a partial state of Arena-Idb that contains a ncRNA. The input ncRNA is labelled with a HGNC symbol that equals the identifier assigned to the ncRNA present in the partial state. Instead, there is no identifier that can match the gene symbol. The input information also report a biological production of the ncRNA from the given gene. The gene is added to the partial knowledge, the two ncRNAs are matched, and the biological relation is flushed

**Fig. 4:**
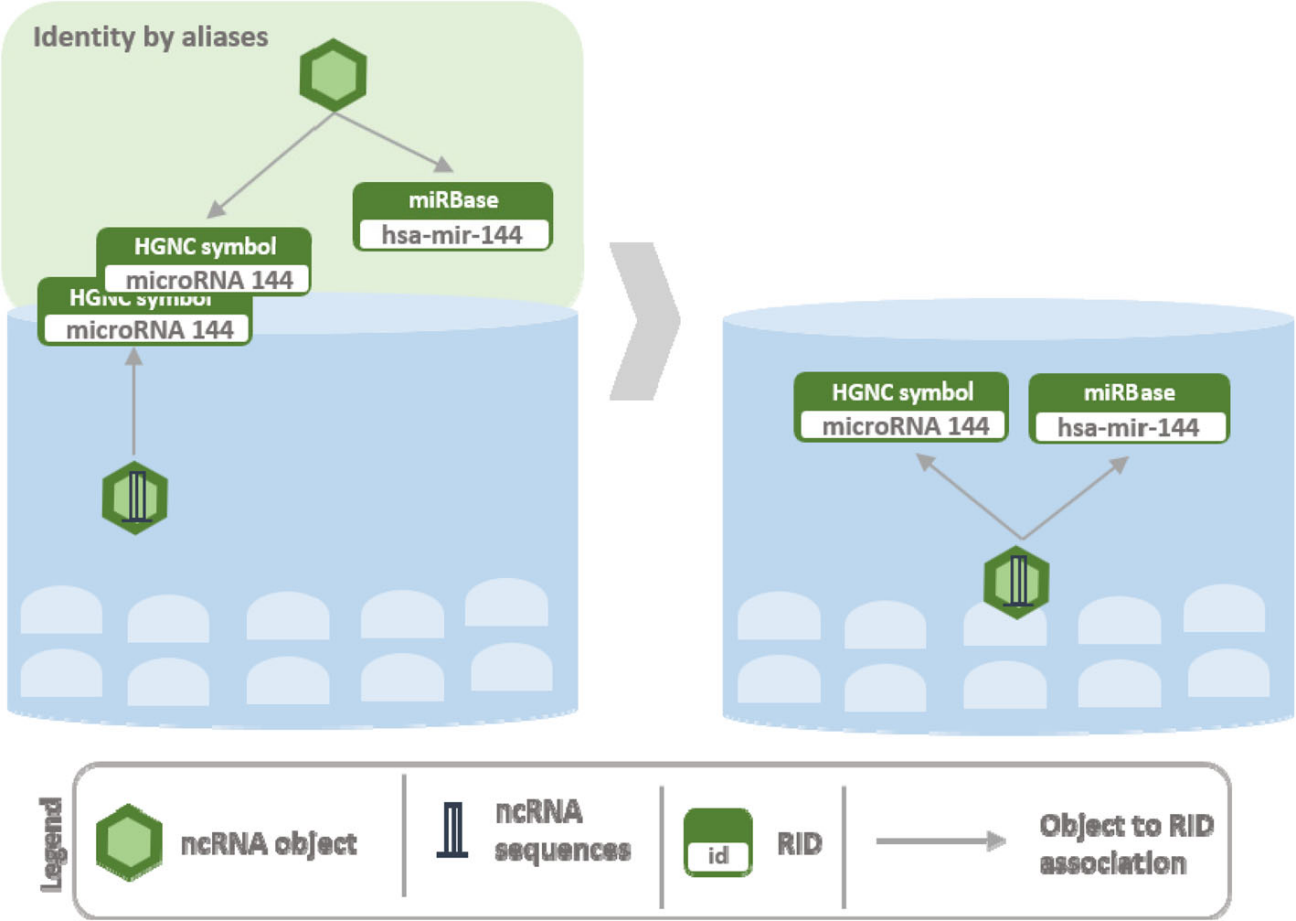
Arena-Idb integration process: indetity by aliases, example 2 The Figure gives an example of integration that does not add any new object to the current knowledge, instead it extends the set of aliases linked with the existing object. The input ncRNA has no assigned sequence, thus a recognition by sequences is not available. The HGNC symbol is used to recognize the identity of the two ncRNAs, and the miRBase identifier is added to the list of the aliases linked with the ncRNA

**Fig. 5:**
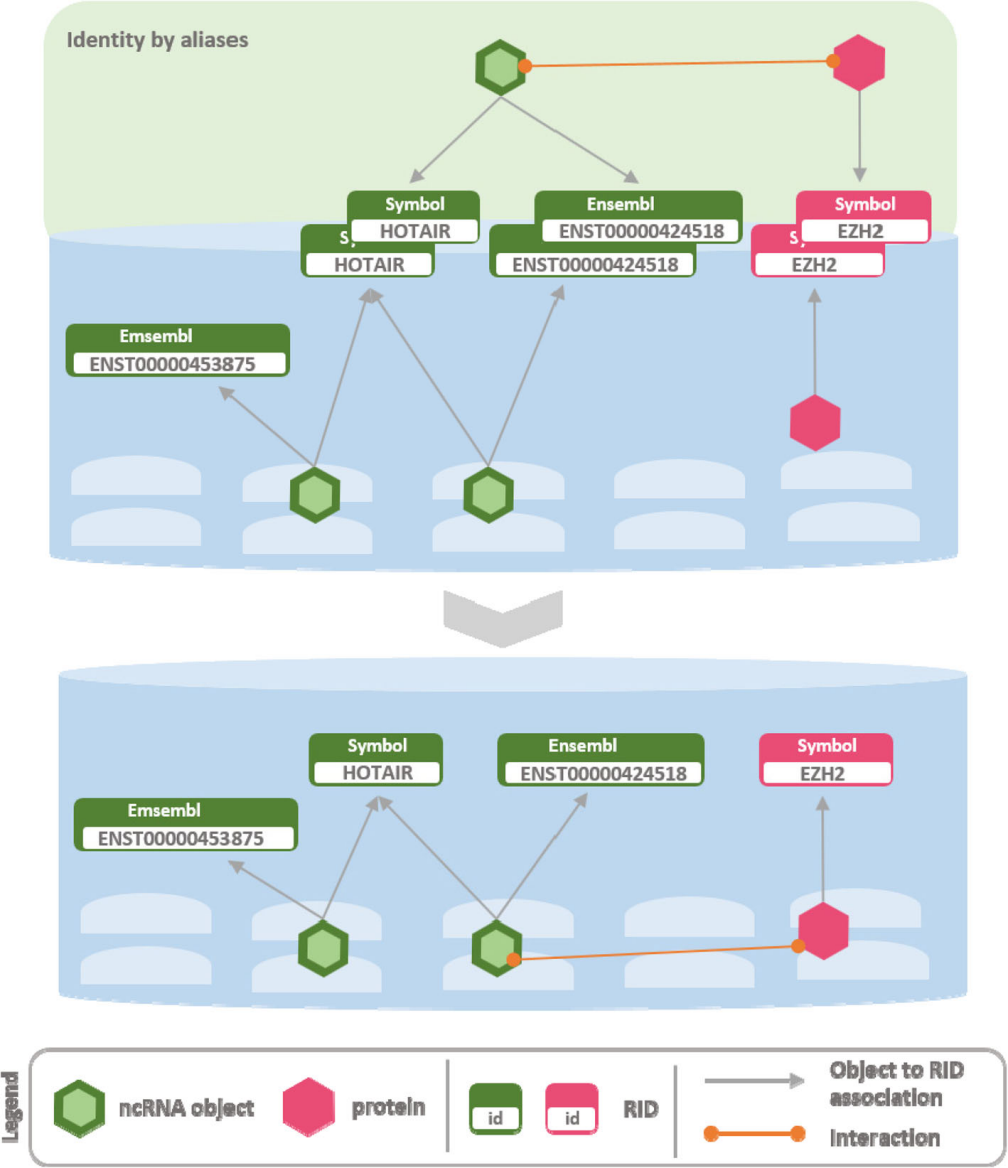
Arena-Idb integration process: identity by aliases, example 3 The Figure reports an example of integration of a ncRNA and a protein into a partial state of Arena-Idb. The ncRNA is labelled with two identifiers, a symbol and an Ensembl ID, and the protein is labelled only with a symbol. A interaction between the two objects is reported. In this case, two ncRNAs are already present in the partial knowledge. They are alternative transcripts of the HOTAIR gene, thus they are labelled with the symbol *HOTAIR*. However, the two ncRNAs can be distinguished by the specific Ensembl identifier linked with them. The integration procedure recognizes the identity of the input ncRNA with one of the two already present in the partial state by means of the Ensembl identifier. On the contrary, the input protein is directly mapped to a protein already in the partial state since no alias ambiguity arises. Finally, the biological interaction is flushed to the final knowledge base

The construction of Arena-Idb is realized through a series of sequential steps that go from the collection of data from different ncRNA and interaction databases to the mining and integration of data for the construction of heterogeneous interaction networks. An overview of the process developed for the integration of input data sources is shown in Fig. 1. A initial non-redundant collection of ncRNAs is built by performing object recognition via sequence identity. Interaction sources, that also contain other types of objects, are integrated by cross-link identity recognition. The result of the integration contains information about the objects, the interactions between ncRNAs and integrated objects and biological products from genes to ncRNAs. Figures 2, 3, 4, and 5 give the details of the integration process summarized into four steps. We first describe how data are extracted and represented in Arena-Idb, than we describe each integration step sequentially.

### Data content

The Arena-Idb data storage is implemented using two different Database Management Systems (DBMS): i) a Relational DBMS, MySQL release 5.5, and ii) a Graph DBMS, neo4J community edition 3.1.3. The MySQL database stores data about names, annotations and sequences and it is used to efficiently query ncRNAs and to optimize the retrieval of associated annotations and sequences information. The Graph DBMS efficiently handles the construction and visualization of the networks of thousands of biological entities (nodes) and relations (edges). We use the relation part of the data storage also to facilitate the integration in Arena-Idb of new data sources (often released as relational DBMSs). We developed specific procedures in Cypher Query Language for the data porting from relational DBMS to Neo4J which automatically ingest relationships and graph information about alias, multi-resources referencing and biological entities interactions.

Table 2 reports the data sources integrated in Arena-Idb together with further information such as the type of extracted biological entities. To gather data from all sources we implemented customized Extract, Transformation and Load (ETL) procedures for data available in different forms: TSV (Tab-separated values), CSV (Comma- separated values), and Biomart/Ensembl instances that are queried and processed by REST API, R procedures and Pentaho Data Integration (Kettle) scripts (http://www.pentaho.com/product/data-integration).

**Table 2:** List of the database resources with related information extracted and used in Arena-Idb platform. Legend: BI= Basic Information; S=Sequences; CR=Cross references;); ncRNAs (non-coding RNA); pcRNAs (protein coding RNA); G=Gene; Ps=Pseudogene; D=Disease; P=Protein, GO=Ontology, I=Interactions (NN:ncRNA-ncRNA, NM:ncRNA-pcRNA, NG:ncRNA-Gene, NS:ncRNA-Pseudogene, ND:ncRNA-Disease, NO:ncRNA-Others)

Database	Biological Entities extracted	Annotated Information	Description
HGNC [1]	ncRNA, pcRNA, G, D	BI, CR	A curated collection of approved Human Gene Nomenclature
Genecode [39]	ncRNA, pcRNA, G, PS	BI, S	Reference gene annotation and experimental validation for human and mouse.
VEGA/Havana [40]	ncRNA	BI, S	A repository for gene model produced by the manual annotation.
Ensembl [41]	ncRNA	BI, S, CR	Genome browser database for vertebrate with annotate gene.
miRBase[42]	ncRNA	BI, S	Database of of published miRNA sequences and annotation.
RefSeq [43]	ncRNA	BI, S	Collection of integrated, non-redundant and well annotated set of transcript and genomic data.
GtRNAdb [44]	ncRNA	BI, S	Genomic tRNA database.
piRNAbank [45]	ncRNA	BI, S	Resource on classified and clustered piRNAs.
Disease Ontology [46]	D, GO	CR	Database of standardized ontology of human disease.
Circ2Traits [47]	ncRNA, pcRNA, G, D	NN, NM, NG, ND	A comprehensive database of human circRNAs associated with diseases and traits.
HMDD [48]	ncRNA, G, D	NG, ND	A collection of experimentally supported human miRNAs and disease associations.
Lnc2Cancer [49]	ncRNA, D	CR, ND	A manually curated database of experimentally lncRNAs associated with cancer.
LncActDB [50]	ncRNA, D	NN, NG, ND	Database containing a list of lncRNA and mRNA with regulatory roles.
LncRNAdb [51]	ncRNA, G, P	NN, NG, NP	A database of functional lncRNAs.
LncRNADisease [52]	ncRNA, D	NP, ND	A curated DB of lncRNA with diseases.
Mir2diseases [53]	ncRNA, G, D	NG, ND	A manually curated database for miRNA deregulation in human diseases.
MiRandola [6]	ncRNA, D	ND	Collection of extracellular circulating miRNAs and their deregulation in human disease.
miRecords [54]	ncRNA, G	NG	A collection of validate miRNA target interaction with the exclusion of predicted interactions.
miRTarBase [55]	ncRNA, G	NG	A database of experimentally validate miRNA target interactions.
mirSponge [56]	ncRNA, pcRNA, G, Ps, D	NN, NM, NG, NP, ND	Manually curated database of miRNA spanges and ceRNAs.
NONCODE [57]	ncRNA	CR	A database of ncRNA with integrated only the Cross-References.
NPInter [18]	ncRNA, P	NN, NP	Database of experimentally verified interaction between ncRNA and other biomolecules.
PSMIR [58]	ncRNA	NO	A database of potential associations between small molecules and miRNAs.
StarBase [59]	ncRNA, G, P, Ps	NN, NG, NS, NP	A database of miRNA-mRNA interactions.
TarBase [60]	ncRNA, Gene	NG	A database of curated experimentally validate miRNA targets.

Sequence data in Arena-Idb are loaded by using REST Biomart API calls for VEGA/HAVANA and ENSEMBL ncRNAs, by parsing the Genbank entries files (GBFF flat files) downloaded from NCBI FTP using BioJava API calls, and by parsing downloadable fasta formatted files from mirBase, GtRNAdb, and pirnaBank. Tables 3 and 4 report the total amount of entities and interactions, respectively, that result in Arena-Idb at the end of the integration process.

**Table 3:** List of the number of biotypes with alias present in Arena-Idb and the number of their interactions

Name of Biotype	Total
ncRNA	170.919
pcRNA	4.987
Gene	51.599
Pseudogene	16.754
Protein	2.019
Disease	844
Other-Small molecule	1.309

**Table 4:** Number of interactions between different biological classes in the Arena-Idb platform

Interactions type	Total
ncRNA-ncRNA	285.346
ncRNA-pcRNA	455.041
ncRNA-Gene	3.124.380
ncRNA-Pseudogene	24.589
ncRNA-Protein	126.702
ncRNA-Disease	64.278
ncRNA-other	150.535

Arena-Idb stores biological entities according to their biological classes (gene, pseudogene, pcRNA, ncRNA, protein, phenotype, other) and biotype. A biotype is a consensus classification of entities by their physical or functional characteristics, for example the distinction between long non-coding RNAs and microRNAs or circulating RNAs (http://vega.archive.ensembl.org/info/about/gene_and_transcript_types.html).

Biological entities are often reported in multiple sources. Some of them define an internal nomenclature system, called also namespace, and assign new identifiers to entities. Some others use existing identifiers assigned in external namespaces. We refer to those identifiers as RIDs (Reference-ID). More precisely, a RID is a pair of strings, the first one refers to the reference namespace, and the second string reports the identifier within the namespace (for example HGNC:29665). Most reference sources also provide mappings between internal and external RIDs, such mappings are called cross-references.

In Arena-Idb, RIDs are stored apart from entities, and may be linked to multiple entities, possibly with different entity classes. Interactions are stored as tuples containing the internal identifiers of the interacting biological entities, the names and versions of the original data sources, the tools predicting the interactions (if they are not validated), and the PubmedIDs of the scientific articles reporting them together with supporting sentences from the bibliography.

### Identity by sequence: detection of redundant non-coding RNAs by sequence similarity

The first step of the Arena-Idb pipeline integrates sources of non-coding RNA sequences into a non-redundant collection of ncRNA objects. The task is performed by using the Cleanup tool [25], a fast program for removing redundancies from nucleotide sequence databases. Sequences having high grade of identity and overlap, in the same biological biotype, are purged.

Figure 2 shows an input resource providing two ncNRAs with associated sequences *s*_1_ and *s*_2_. The partial collection already contains the ncRNAs having sequences *s*_1_ and *s*_3_. The integration tool recognizes the two ncRNAs having sequences *s*_1_ as the same object, and produces an updated non-redundant collection composed by *s*_1_, *s*_2_, and *s*_3_. The collection of data obtained by merging all the sequence sources is used as base in Arena-Idb for the successive integration steps.

### Identity by alias: detection of redundant entities by RIDs comparisons

RIDs in a namespace are designed to be specific of a given object, and cross-references are supposed to help in mapping entities between different namespaces. However, cross-references do not map every namespace to another, and they may introduce inconsistency and ambiguity. As a result, biological entities may share one or several identifiers, making the task of recognizing them as distinct objects a bottleneck on the integration process. In addition, input source may have a lack of information. Mining procedures in Arena-Idb allow deducing missing data. For example, for entities without reported biological classes, Arena-Idb finds out their classes by searching for entities with a similar set of linked RIDs. Arena-Idb follows an order of resource integration corresponding to the amount of information provided by each source (miRTarBase, HMDD, miR2Disease, miRecords, miRandola, circ2Traits, NPInter, miRSponge, starBase, lncACTdb, Psmir, TarBase, Lnc2Cancer, LncRNADisease, lncRNAdb).

The integration procedures are performed by comparing the sets of RIDs associated with them. For every input entity, if the current collection contains an entity with a comparable set of RIDs, then the input entity is matched to it, otherwise the entity is added up to the collection.

Figure 3 shows two input RIDs having the same label that is *microRNA 144* but associated with objects of different class, a ncRNA and a gene. In the current state of Arena-Idb the RID related to *microRNA 144* is mapped to a ncRNA. Therefore, the input ncRNA and the one already in Arena-Idb are recognized as the same object. On the contrary, the input gene does not have a correspondence in Arena-Idb, thus it is added to it, together with its linked RID. Entities of different classes but having same RIDs are real examples of transcripts named with the same label used for their producer genes. Figure 4 shows the import of a cross-reference linking two RIDs, *microRNA 144* and *hsa-mir-144*, that are referred to the same ncRNA object. The current state of Arena-Idb already contains a ncRNA object labelled with *microRNA 144* but missing of the *hsa-mir-144* RID. The identity by aliases approach implemented by Arena-Idb recognizes the equivalence of the two objects, since they have the same label *microRNA 144* in common, and the integration procedure updates, with the additional RID *hsa-mir-144*, the information linked to the ncRNA.

Figure 5 reports a real example of transcripts sharing one or more RIDs, possibly because they are isoforms of the same gene. The input source contains a ncRNA with two RIDs: *HOTAIR* and *ENST00000424518*. The procedure maps the input entity with the ncRNA having a complete match with the set of aliases of the input ncRNA, while the ncRNA associated to *ENST00000453875* partially overlap the set. Figure 5 gives also an example of cross-references. Once entities of an input source are mapped to those already contained in the database, the information regarding interactions and additional cross-references is added to Arena-Idb. As a result, the step unifies the plenty of integrated sources and provides a higher comprehensive view of the currently known information regarding interactions in which ncRNAs are involved.

Finally, during the integration, customized procedures regarding miRNAs and disease names are applied. Arena-Idb adds, to the miRNA entities, additional RIDs that refers to miRNA genes (see http://www.mirbase.org/help/nomenclature.shtml). Regarding phenotype entities, in presence of RIDs containing parenthesis, names are split into two or more identifiers. Arena-Idb also defines a set of regular expressions to express all extracted RIDs identifiers (e.g., HGNC:[0-9] refers to HGNC IDs). Since RIDs may lack of reference source names, the integration procedure approximately matches the incomplete RID against a set of regular expressions in order to assign the correct namespace.

### Detection of primary names

A final step of integration is performed to assign a single representative RID, called primary name, to every biological entity. The algorithm extracts subsets of entities belonging to the same biological class and sharing at least one RID. In order to choose the primary names, the algorithm takes into account two properties regarding RIDs. First, it defines the following order of trustiness resources: miRBase, VEGA, RefSeq, Ensembl, GtRNAdb, piRNABank, snoRNABase, Entrez, and all the other not listed resources have the same preference order. Second, it counts the number of entities that are linked to a given RID. Identifiers with fewer entities are preferred. The described combinatorial approach is hard to solve cause every possible combination of RIDs to entities must be scanned. Since, similar combinatorial problems are well-known in literature, such as the “stable marriage problem”, we represent entities and RIDs in a bipartite network and apply heuristics to reduce the computational time needed to find a solution for the mapping. Briefly, entities with the fewest number of RIDs linked to them are accounted firstly, and the sets of their RIDs are sorted by the above precedence’s list.

### Data update

Data update is performed by re-running globally or partially the ETL procedures. More precisely, we can summarize the database population procedure into two main steps. In the first step, semi-automatic ETL procedures (tailored to each input sources) gather data from external primary sources, producing a homogeneous representation of input resources and merge it into a single knowledge base. In the second step, the external interaction sources are parsed and all the interactions among the mates are built. Therefore, a main update of Arena-Idb involves the execution of all the ETL procedures to build the database from scratch. However, updating a single external source only consist of the execution of the scripts related to that source in the first and second phase. Furthermore, the normalization performed by the first ETL phase allows to add new external resources to the system without substantial modification of the overall procedure, the database maintainer can execute only the ETL script related to the new source using the developed ETL as template.

## Results

The Arena-Idb provides an easy-to-use graphical web interface and graphical visualization to facilitate the retrieval of ncRNAs interactions. The Graphical User Interface (GUI) has been developed as JAVA Web Application in Java Platform Enterprise Edition - Java EE. It uses jQuery/jQuery-UI framework JavaScript on the client layer, Java servlets and JavaServer Pages (jsp) on the server layer. The web application is deployed in a Tomcat web server (https://tomcat.apache.org). The Hibernate ORM (Object Relational Mapping, http://hibernate.org/orm/) has been adopted to implement the communication between the data layer (MySQL and Neo4j) and the Web Application. It also provides a framework for mapping an object-oriented domain model to relational and graph databases enabling us to handle the data layer as objects in the web pages.

Arena-Idb provides two modes to access to data, Search and Browser. Browser lists in a tabular mode all pairs of interacting entities in Arena-Idb reporting their tuples of information (as described in Data content section). User can browse by RNA-RNA, RNA-gene, RNA-Protein, and RNA-Disease interaction.

**Fig. 6:**
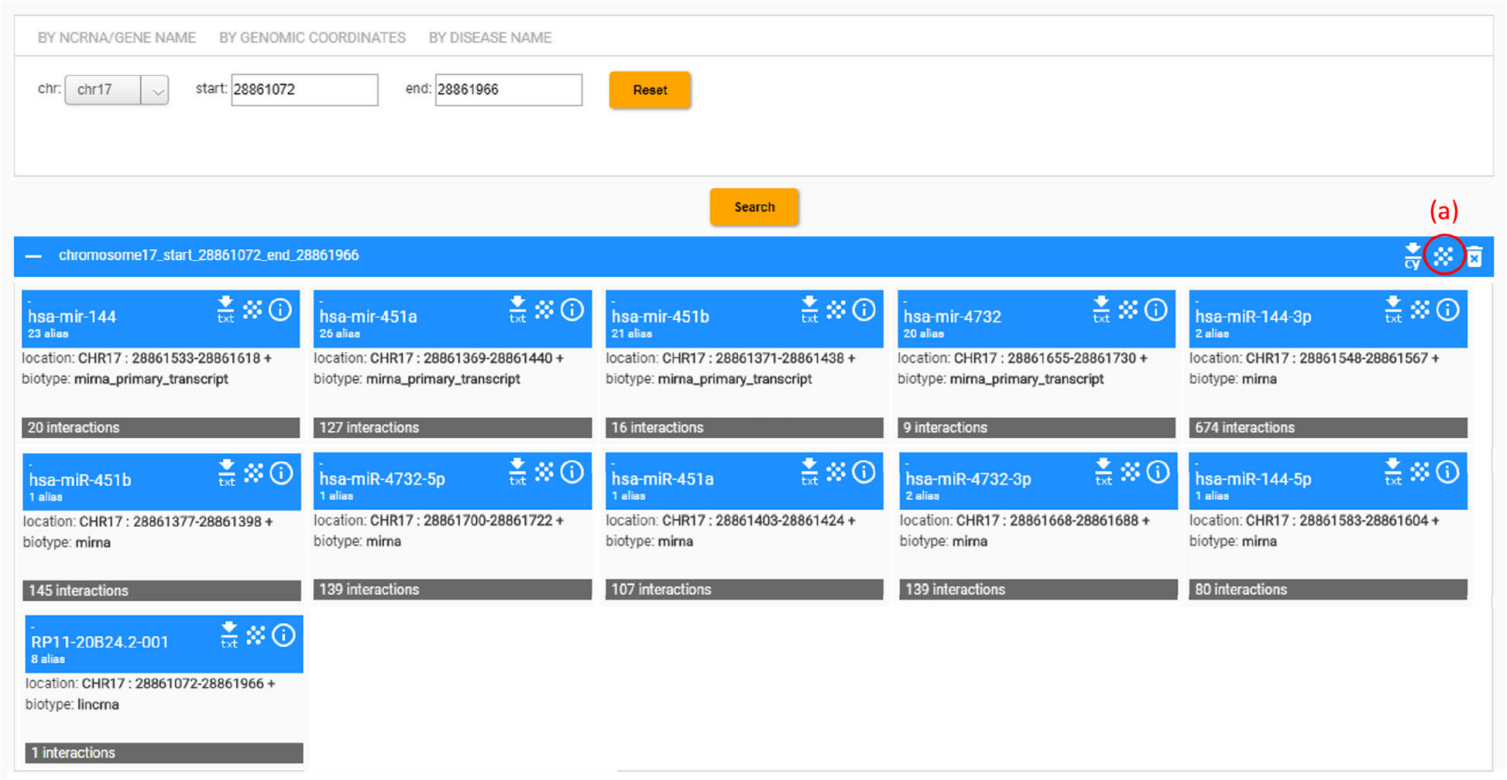
Arena-Idb Search web interface. Search is performed by ncRNA or gene name, by genomic coordinates, or by disease name. Here the search is performed by looking at 28861072 to 28861966 positions in chromosome 17. Results are memorized on query named ’chr17-q’. Arena-Idb returns 11 entities retrieved in the desired chromosome location. Each card shows the subnetwork of the retrieved entity. User can click on the buttons in top right (**a**) of the query result frame to visualize a global network obtained by merging all possible interactions among nodes in the retrieved subnetworks

The Search mode allows to retrieve ncRNAs using the following criteria: by ncRNA/gene name, by genomic coordinates, and by disease name (see Fig. 6). When one starts typing ncRNA/gene name or disease name into the search box, suggested ncRNA/gene or disease names are displayed in the list box. The end user chooses one of the names associated to the biological entity from the list box. In order to use the search by genomic coordinates the user chooses the number of the chromosome and the starting and ending positions of the desired region in that chromosome. All run queries are listed and can be retrieved in MY SEARCH section.

The results of the search are given as a set of ncRNA cards (see Fig. 6). The user can click on the icons in the top of each card to: (i) show in tabular form a detailed page reporting information such as genomic locations, synonyms, sequence, and the list of interactions; (ii) to show interactively the interactions represented graphically as a network; and (iii) to download the interactions in a format compatible with advanced network mining and visualization platforms such as Cytoscape (http://www.cytoscape.org/) or as text file in FASTA and TSV format.

Furthermore, Arena-Idb creates a whole network of interactions by merging all the retrieved entities and adding to the network all possible interactions stored in Arena-Idb among them. This can be visualized by clicking on the icon on the top right of query result bar, see Fig. 6a. The merging can also be done gradually under the guide of the user by adding one at a time interacting entity or type of interaction. The obtained global (merged) network can be downloaded in tabular format as described above.

Networks can be filtered by deselecting entity types (protein, ncRNA, pcRNA, disease, gene and other) and thus removing all nodes of such types and their edges. The edges are associated to scores representing the number of resources reporting such interactions. Scores range from 1 to the total number of integrated resources. A dark grey corresponds to a high score. Clicking on a node, a tooltip window displays all the associated RIDs (name and aliases), while clicking on the arch a tooltip with the score number and the type of interaction is displayed. User can navigate inside the displayed network by zooming in/out. Clicking on a node, Arena-Idb also highlights the node itself and its neighborhood.

## Discussion

**Fig. 7:**
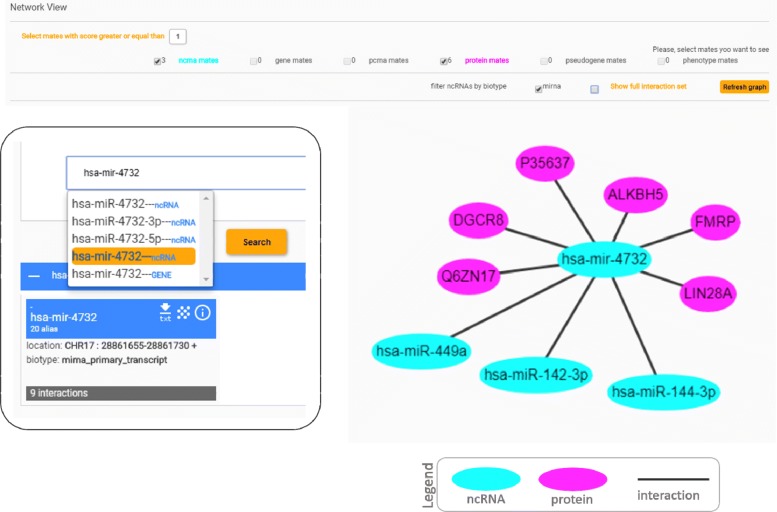
Arena-Idb interaction network visualization of hsa-mir-4732. The Figure shows the network visualization interface displaying the interactions regarding the miRNA primary transcript *hsa-mir-4732*. The transcript has a total of 9 interactions, three with other miRNAs and 6 with proteins. In the network visualization mode different colours are used to represent diverse biological entities (i.e. nodes): Protein (fuchsia), ncRNA (cyan), pcRNA (green), disease (purple), gene (orange), and other (pink). Arena-Idb also contains information about the generic miRNA *hsa-miR-4732* (not in its primary transcript form), its 3’ and 5’ transcription and the corresponding gene. The box on the left side of the image shows the right selection of the transcript regarding the network example from the search interface

As an example of Arena-Idb usefulness we describe the case of hsa-mir-4732. Figure 7 shows the interaction network extracted by Arena-Idb searching for hsa-mir-4732. The interaction network indicates that hsa-mir-4732 is related to hsa-miR-449a, hsa-miR-142-3p and hsa-miR-144-3p. Looking at the genomic location of this microRNAs we found that hsa-miR-144, and hsa-mir-4732 are transcribed as a polycistronic gene. Many of the known miRNAs are distributed across chromosomes either individually or in cluster, in which two or more miRNA genes are located within a short distance on the same segment of a chromosome. The miRNA cluster arose through a complex history of duplication and loss of individual members as well as duplication of the entire cluster. Several studies suggest a role of the miRNA-144 cluster in the complex regulation of the expression of genes involved in different diseases and relationships in the hsa-mir-4732 network extracted by Arena-Idb found meaningful evidence in the literature [27–32].

**Fig. 8:**
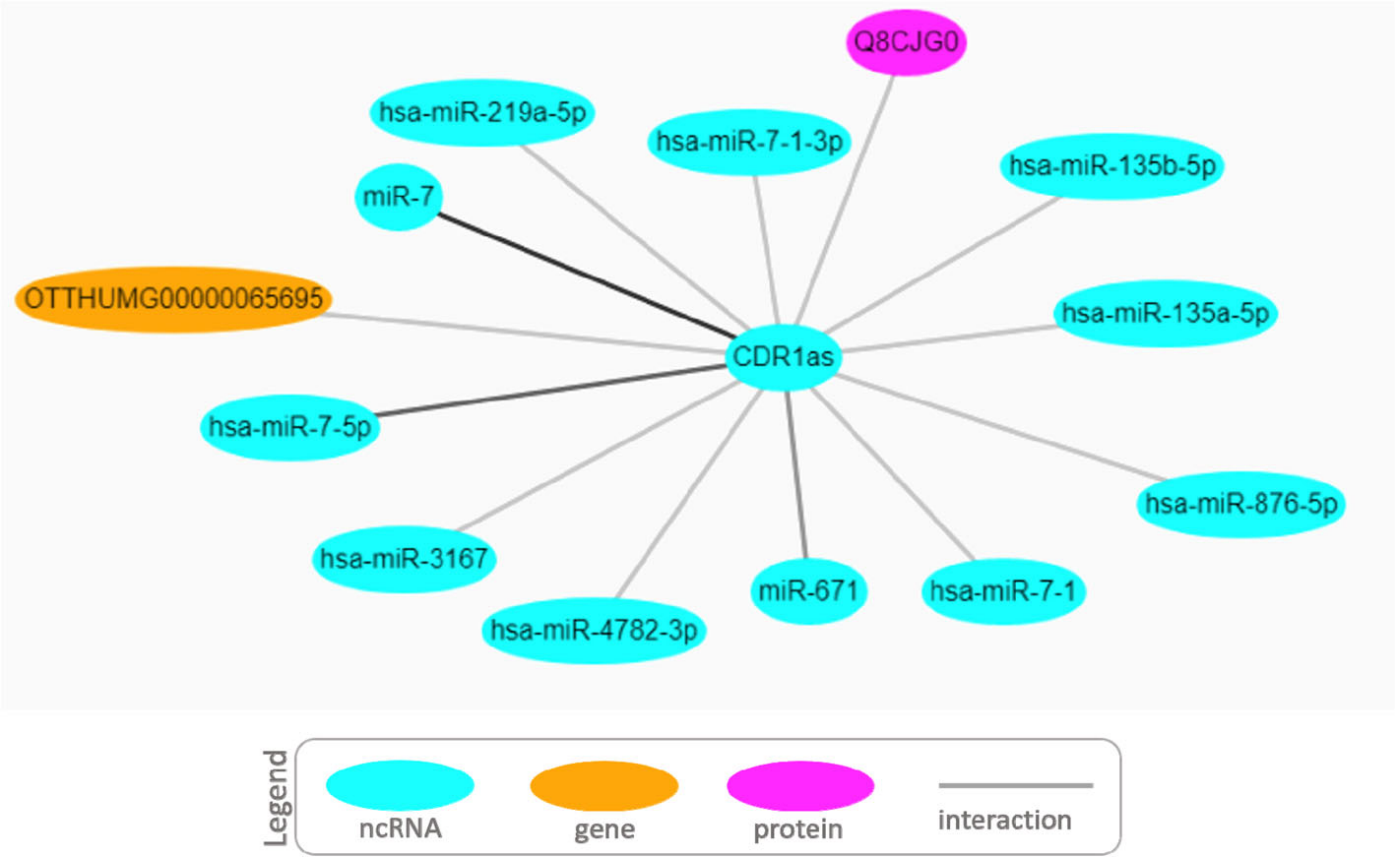
Arena-Idb interaction network visualization of CDR1as. In [35] authors show that CDR1as causes miRNAs deregulation and affects brain function, in particular miR-7 and miR-671. Arena-Idb is able to retrieve and easy visualizes the strong cited relations, suggesting also further entities to investigate

An additional example is represented by a circular RNAs (circRNAs), CDR1as. Genome-wide analyses have identified a large number of abundant circRNAs that represents a recent addition to the growing list of ncRNA classes [33, 34]. CircRNAs can arise from exons (exonic circRNA) or introns (intronic circRNA) and act as miRNA sponges thus playing a role in mediating miRNA targeting. The Fig. 8 shows the interaction network extracted by Arena-Idb for CDR1as. In particular it shows a strong relationship with miR-7 (score: 4, sources: lncrnadb) and miR-671 (score: 2, source: lncrnadb), two miRNAs whose activity is affected by CDR1as, as reported by the Kjems laboratory [35].

## Conclusion

ncRNAs are crucial for many biological processes. Despite many studies have indicated the importance of ncRNAs in different tissues and diseases, little is known about their biological functions and interactions. New complex interactions among ncRNAs, and between ncRNAs and diseases, have emerged [36–38].

Research on the functional and clinical role of ncRNAs in molecular biological processes with implications in human diseases has exploded since they were discovered a decade ago, implying a proliferation of online resources to store ncRNAs and their interactions. These databases are frequently developed only in the contest of one or few types of ncRNAs, and they miss in providing an integrated vision of the relationships between different ncRNA classes and other entities. The advantages that Arena-Idb provides to end-users is the availability of a framework for reconstruction of networks of ncRNA interactions with other biological entities and diseases, that can be modelled on-demand and filtered for more specific interactions depending on the users needs. Another important feature is the minimization of ambiguities that in the case of the ncRNAs represents a big problem due to missing effective standards for their nomenclature and heterogeneity of resources used. Moreover, ArenaIdb can be downloaded as a whole system to customize additional resources integration. All together these features make of ArenaIdb an exhaustive and useful reference for user to explore at large any type of interaction and to discover unforeseeable functional role of not yet characterized ncRNAs.

## Abbreviations

API: Application programming interface
ceRNA: Competitive endogenous RNA
CSV: Comma-separated value
DBMS: DataBase management system
ETL: Extract, transform and load
exRNA: Extracellular RNA
GUI: Graphical user interface
lncRNA: Long non-coding RNA
miRNA: MicroRNA
ncRNA: Non-coding RNA
piRNA: PIWI-interacting RNA
REST: REpresentational sate transfer
RID: Reference identifier
RNA: RiboNucleic acid
sncRNA: Small non-coding RNA
siRNA: Small interfering RNA
snoRNA: Small nucleolar RNA
snRNA: Small nuclear RNA
TSV: Tab-separated value
